# Finite Element Analysis-Based Vertebral Bone Strength Prediction Using MDCT Data: How Low Can We Go?

**DOI:** 10.3389/fendo.2020.00442

**Published:** 2020-07-28

**Authors:** Nithin Manohar Rayudu, Karupppasamy Subburaj, Kai Mei, Michael Dieckmeyer, Jan S. Kirschke, Peter B. Noël, Thomas Baum

**Affiliations:** ^1^Engineering Product Development (EPD) Pillar, Singapore University of Technology and Design (SUTD), Singapore, Singapore; ^2^Department of Radiology, Perelman School of Medicine, University of Pennsylvania, Philadelphia, PA, United States; ^3^Department of Diagnostic and Interventional Neuroradiology, Klinikum rechts der Isar, Technische Universität München, Munich, Germany

**Keywords:** multidetector computed tomography, bone strength, finite element analysis, osteoporosis, dose reduction

## Abstract

**Objective:** To study the impact of dose reduction in MDCT images through tube current reduction or sparse sampling on the vertebral bone strength prediction using finite element (FE) analysis for fracture risk assessment.

**Methods:** Routine MDCT data covering lumbar vertebrae of 12 subjects (six male; six female; 74.70 ± 9.13 years old) were included in this study. Sparsely sampled and virtually reduced tube current–based MDCT images were computed using statistical iterative reconstruction (SIR) with reduced dose levels at 50, 25, and 10% of the tube current and original projections, respectively. Subject-specific static non-linear FE analyses were performed on vertebra models (L1, L2, and L3) 3-D-reconstructed from those dose-reduced MDCT images to predict bone strength. Coefficient of correlation (*R*^2^), Bland-Altman plots, and root mean square coefficient of variation (RMSCV) were calculated to find the variation in the FE-predicted strength at different dose levels, using high-intensity dose-based strength as the reference.

**Results:** FE-predicted failure loads were not significantly affected by up to 90% dose reduction through sparse sampling (*R*^2^ = 0.93, RMSCV = 8.6% for 50%; *R*^2^ = 0.89, RMSCV = 11.90% for 75%; *R*^2^ = 0.86, RMSCV = 11.30% for 90%) and up to 50% dose reduction through tube current reduction method (*R*^2^ = 0.96, RMSCV = 12.06%). However, further reduction in dose with the tube current reduction method affected the ability to predict the failure load accurately (*R*^2^ = 0.88, RMSCV = 22.04% for 75%; *R*^2^ = 0.43, RMSCV = 54.18% for 90%).

**Conclusion:** Results from this study suggest that a 50% radiation dose reduction through reduced tube current and a 90% radiation dose reduction through sparse sampling can be used to predict vertebral bone strength. Our findings suggest that the sparse sampling–based method performs better than the tube current–reduction method in generating images required for FE-based bone strength prediction models.

## Introduction

Osteoporosis is a skeletal disorder that occurs due to excessive reduction in bone mass and microstructural degeneration (1). The changes induced in bone morphology and bone density by osteoporosis are often undetected until the occurrence of fragility fractures. Lumbar compression fractures are one of the most common types of vertebral fractures that occur due to osteoporosis, and these fractures often result in a substantial increase in morbidity, disability, dependency, and cost of management (2, 3). These fractures significantly impact the quality of life because most patients do not get to return to their previous functional status to perform activities of daily living (4). Thus, the assessment of bone health at an early stage of the disease is critical in terms of treatment initiation and fracture prevention.

At present, dual-energy X-ray absorptiometry (DXA)–based aerial bone mineral density (aBMD) measurements (*T*-score and *Z*-score) are considered as the gold standard for clinical osteoporosis diagnosis. However, this method has <50% accuracy in identifying patients at risk of having bone fracture (5). The significant overlap in the BMD values observed in patients with and without osteoporotic fractures strongly suggests that BMD alone is insufficient in assessing bone health (6). Overall bone health is affected by a multitude of factors, including strength, morphology, density, geometry, and spatial bone mass distribution (7–9). In this context, analyses of the 3-D microstructures of bone and their influences on overall bone health are warranted. Also, the complex anatomical structure of bone tissue emphasizes the importance of considering the critical interaction between different structural elements of the bone while assessing bone fracture risk (10). Quantitative computed tomography (QCT) imaging has been used in place of DXA to measure volumetric BMD from attenuation values, calibrated using a phantom, in assessing bone quality and condition (11–14).

Subject-specific finite element (FE) models have been used in solving biomechanically related clinical problems, including bone strength predictions (15–18). In FE analysis, realistic 3-D patient-specific models of tissues and bones are reconstructed from radiological image data, material properties are applied to an FE meshed model based on attenuation values [Hounsfield units (HU)], and then boundary and loading conditions are applied to obtain mechanical, structural, and fracture characteristics of them. Studies have shown that FE-based analysis can reliably predict bone strength (15) and fracture risk (5). Volumetric BMD with FE-predicted bone strength can predict the fracture risk more accurately compared to DXA-based BMD or FE-predicted bone strength alone (19). Although the evaluation of bone strength with FE analysis could provide a better understanding of the osteoporotic fracture risk, it has not reached patient care or been implemented as routine clinical protocol due to the concern of excessive radiation exposure, processing time, and associated cost (20).

In this work, we focus on the first challenge, excessive radiation risk associated with multiple scanning needed for disease and treatment monitoring. The use of MDCT scans for routine clinical diagnosis and monitoring of patients with a risk for osteoporotic fracture has increased (21). In modern CT scanners, the effective dose for the lumbar spine scan is between 5.6 and 10.0 mSv (21, 22). The effective dose depends on different scanning parameters, including tube current, number of projections, scan time, scan range, body part, and tube potential. In the literature, researchers have explored different strategies, including tube current reduction and sparse sampling (acquiring fewer projections during scanning), to reduce the effective dose for clinical diagnosis (23, 24). These dose-reduction strategies increase image noise and reduce signal-to-noise ratio and image quality with standard reconstruction algorithms (15). Advanced image reconstruction techniques, such as physics-based statistical iterative reconstruction (SIR), have shown potential in increasing image quality of these low-dose images to the level comparable to the standard dose images (25, 26). However, the effect of these two prominent dose-reduction strategies in predicting vertebral bone strength using finite element analysis has not been studied yet.

Thus, the scope of the current work was to study and compare the effect of tube current reduction and sparse sampling–based dose reduction strategies on the vertebral bone strength calculation using finite element methodology. To achieve the purpose stated above, we set out to investigate the following objectives:

1. Analyze and compare the FE-predicted failure load values at different reduced dose levels achieved through the tube current–reduction method with the value at the standard dose level.
2. Analyze and compare the FE-predicted failure load values at different reduced dose levels achieved through the sparse sampling method with the value at the standard dose level.
3. Compare the FE-predicted failure load values at different reduced dose levels through both dose reduction strategies.

## Materials and Methods

**Figure 1:**
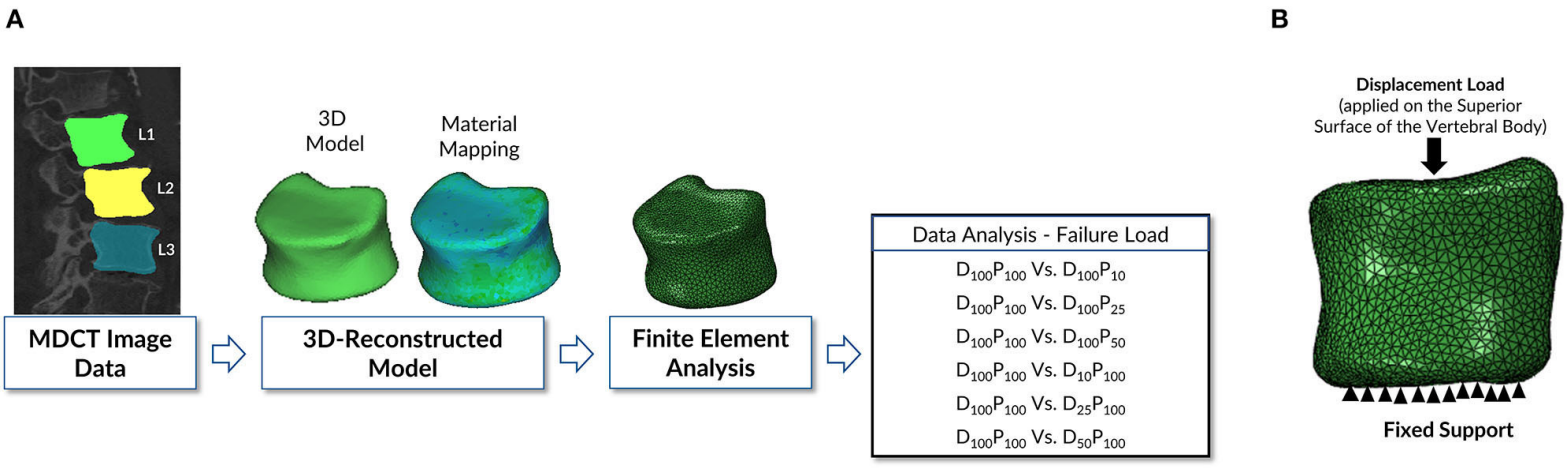
**(A)** Schematic representation of the finite element modeling and analysis methodology followed in this study. The vertebrae were delineated on the images acquired to generate a 3-D model of the geometry to be used in the downstream finite element analysis protocol to predict bone strength. **(B)** Loading and boundary conditions applied in performing the finite element analysis of vertebra. Fixed support represents the zero displacement in all directions at the inferior surface of the vertebral body. Displacement load was applied on the superior surface of the vertebral body to predict the failure load.

The methodology followed to study the objectives mentioned above is shown in Figure 1A. The methodology in this computational study has four major subsections: MDCT data acquisition; data processing; 3-D reconstruction of vertebral bone models from the images; finite element modeling and analysis, including meshing, realistic material properties mapping, applying loading and boundary conditions, and sensitivity analysis; and data analysis.

### Subjects

A total of 12 subjects (six males and six females, age 74.70 ± 9.13 years) who underwent routine abdominal MDCT were retrospectively included in this study. Subjects with bone changes such as hematological or metabolic bone disorders, bone metastases aside from osteoporosis, and vertebral fractures were excluded. For that purpose, clinically available data and previous imaging studies were thoroughly analyzed. The local institutional review board has reviewed thoroughly and approved the current study.

### MDCT Imaging

A 256-row MDCT scanner (iCT; Philips Healthcare, Best, The Netherlands) was used for acquiring all MDCT scans. A reference phantom (Mindways Osteoporosis Phantom, Austin, TX, USA) was placed under the scanner mat for all the subjects during scanning. The tube voltage is kept constant at 120 kVp. During each scan, the scanner implicitly modulated the tube current (min: 200 mA, max: 400 mA). The volumetric CT dose index (CTDIvol) was varied from 2.3 to 13.6 mGy in all subjects.

### Low-Dose Simulations

We used two different approaches to simulate low-dose scans. First, an experimentally validated simulation tool is used in the current study to obtain low-dose data measurements as if the scan data was generated at 50% (D_50_P_100_), 25% (D_25_P_100_), and 10% (D_10_P_100_) of the standard radiation dose applied with respected clinical protocols (27). For accounting electronic noise accurately, detector gain is taken into consideration. The obtained result is very similar to reality, especially for ultra-low tube current (23). Second, another set of low-dose data measurement was obtained by applying the sparse sampling–based method by considering only every second, fourth, and 10th projection angles (D_100_P_50_, D_100_P_25_, and D_100_P_10_, respectively) and removing the remaining projections from the raw data.

### Statistical Iterative Reconstruction (SIR)

All the acquired MDCT scans were reconstructed using the SIR algorithm. The SIR is performed by ordered subset separable paraboloidal surrogate (28) with a momentum-based accelerating approach (29). Based on the previous studies, a proper regularization level is used to optimize image quality at low doses (23, 30). For the subjects, the reconstruction field view of 500 × 500 mm^2^ is considered. The axial slice thickness was kept at 0.30 mm, and no overlapping was observed in adjacent slices. At the MDCT rotation center, the resolution of the images was 0.625 mm, which was the same as the actual collimator's detector width.

### Finite Element Modeling

**Figure 2:**
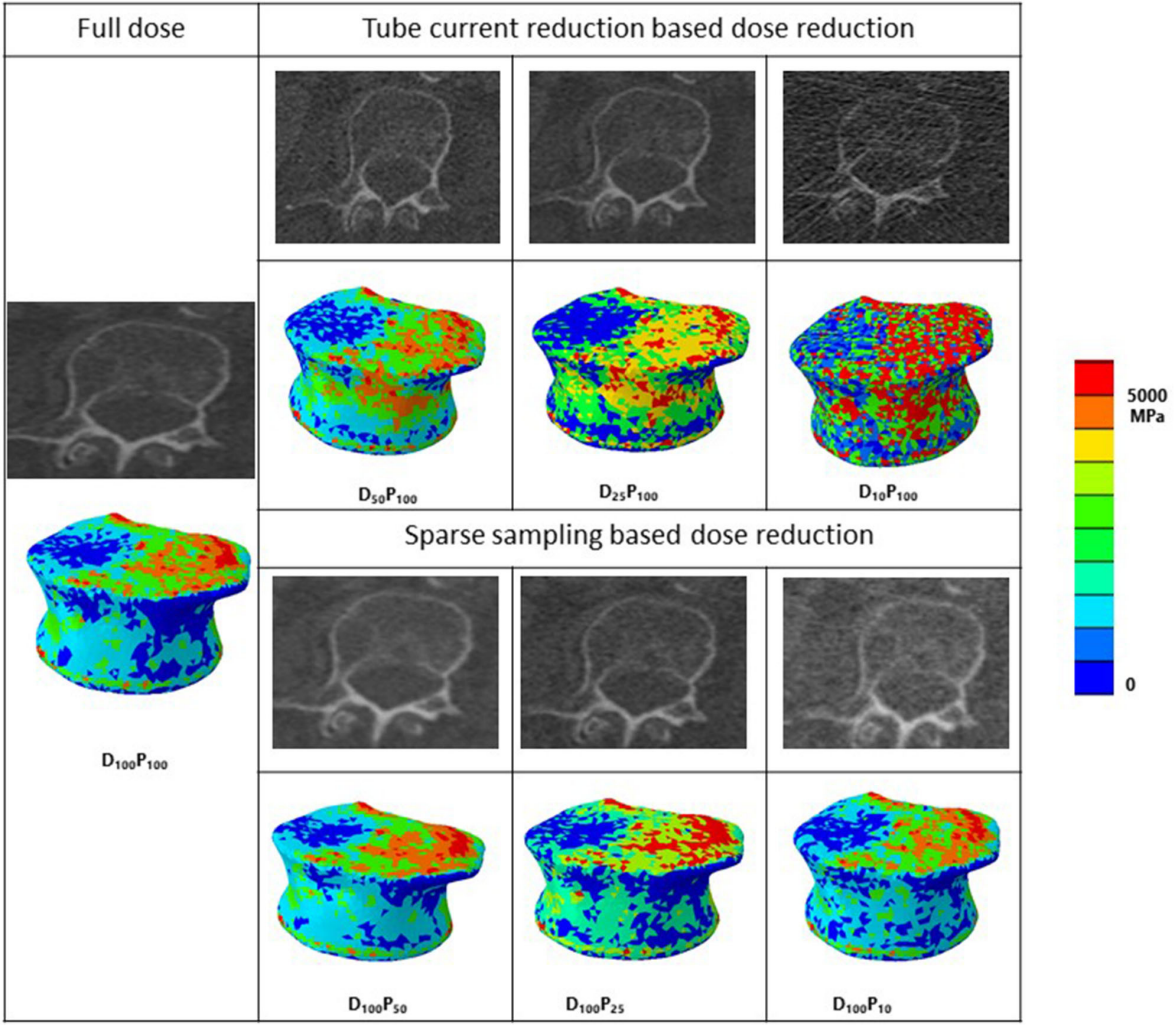
MDCT radiological images along with corresponding representative 3-D contour plots of Young's modulus distribution along the axial direction after material mapping at different dose levels in the vertebra. Red color region shows the maximum, whereas blue color region shows the minimum Young's modulus values in the bone. D, tube current reduction–based dose reduction; P, Sparse sampling–based dose reduction.

Segmentations of the vertebrae L1 to L3 were performed by a radiologist using the Medical Imaging Interaction Toolkit (MITK; www.mitk.org) software program. The MDCT images at different dose levels and segmentation masks of those vertebrae were imported to the 3-D medical image processing software, Mimics (Materialize NV, Leuven, Belgium), for downstream analysis, including 3-D anatomical model reconstruction and material mapping based on attenuation values. Three-dimensional geometric models of those vertebrae were imported in a 3-Matic software program (Materialize NV, Leuven, Belgium) to generate finite element mesh. In the current study, the linear tetrahedral element (C3D4 in the Abaqus element library) is used for meshing. For maintaining the computational accuracy, a mesh sensitivity analysis was done by changing the element's maximum edge length (1.5–3 mm with an increment of 0.25 mm). The analysis showed that the 2-mm element edge length produced a mesh size–independent solution based on vertebral failure load convergence. The same element size was used in all the developed finite element models for further analysis. Once the meshing was performed, material properties of the vertebra then mapped onto the finite element mesh. Table 1 shows the density (ρ)—HU units—elastic modulus (E) material mapping relations used in the current study. Figure 2 shows the MDCT image and mapped axial Young's modulus distribution of the vertebra at different dose levels.

**Table 1:** Vertebral bone material (density - HU - modulus) mapping relations used in the current finite element study (18, 40–42).

**Property**	**Mapping relations**
Apparent density (ρ_app_)	ρ_app_ = 47 + 1.122 * HU HU- Hounsfield unit
Ash density (ρ_ash_)	ρ_app_= 0.6 *ρ_ash_
Elastic modulus (E)	E_z_ = −349 + 5.82 *ρ_app_ E_x_= E_y_ = 0.333 E_z_ Z- axial direction of the vertebra
Shear modulus (G)	G_xy_ = 0.121 E_z_ G_xz_ = G_yz_ = 0.157 E_z_
Maximum principal stress limit (σ)	σ = 137 *ρ_ash_^1.88^, ρ_ash_ <0.317 σ = 114 *ρ_ash_^1.72^, ρ_ash_ > 0.317
Plastic strain (ε_AB_)	ε_AB_ = −0.00315 + 0.0728 ρ_ash_
Minimum principal stress limit (σ_min_)	σ_min_ = 65.1 *ρ_ash_^1.93^

### Failure and Displacement Load Analysis

The meshed and material-mapped model is uploaded to commercial finite element analysis software Abaqus ver. 6.14-2 (SIMULIA, Providence, RI, USA) for further processing. In the current study, the vertebra is analyzed in the compression loading configuration (15, 31). The vertebra's bottom surface was constrained in all directions, and an axial displacement load was applied on the superior surface of the vertebra as shown in Figure 1B. Transversely isotropic properties were given to the vertebra, and the failure load was calculated. The peak of the force-displacement curve was considered as the bone strength in the current study. The FE methodology that was used in the current work was experimentally validated in our previous work (15–17, 32).

### Statistical Analysis

Microsoft Excel, Version 16.27 (2019) (Microsoft Corporation, Redmond, Washington, USA) and IBM SPSS Statistics for Windows, version 25.0 (IBM Corp., Armonk, New York, USA) were used for performing all statistical data analyses. All tests were performed using a two-sided 0.05 level of significance. The distributions of failure load were plotted and examined. The coefficient of correlation (*R*^2^) and linear regression models were used for calculating the variations in the failure loads obtained at reduced dose levels (D_100_P_50_, D_100_P_25_, D_100_P_10_) vs. standard dose level (D_100_P_100_) as a reference for assessing the effect of dose reduction through sparse sampling on the FE-predicted failure load. For calculating the effect of dose reduction through tube current reduction on the FE-predicted failure load, we calculated the coefficient of correlation (*R*^2^) for different dose levels [(D_50_P_100_, D_25_P_100_, D_10_P_100_) vs. standard dose level (D_100_P_100_)]. Also, root mean square error coefficient of variation (RMSCV) in percentage is calculated, and Bland-Altman plots (33) were generated to assess the spread of the FE-predicted failure load values.

## Results

### Effect of Sparse Sampling–Based Dose Reduction on FE-Predicted Vertebral Failure Load

**Figure 3:**
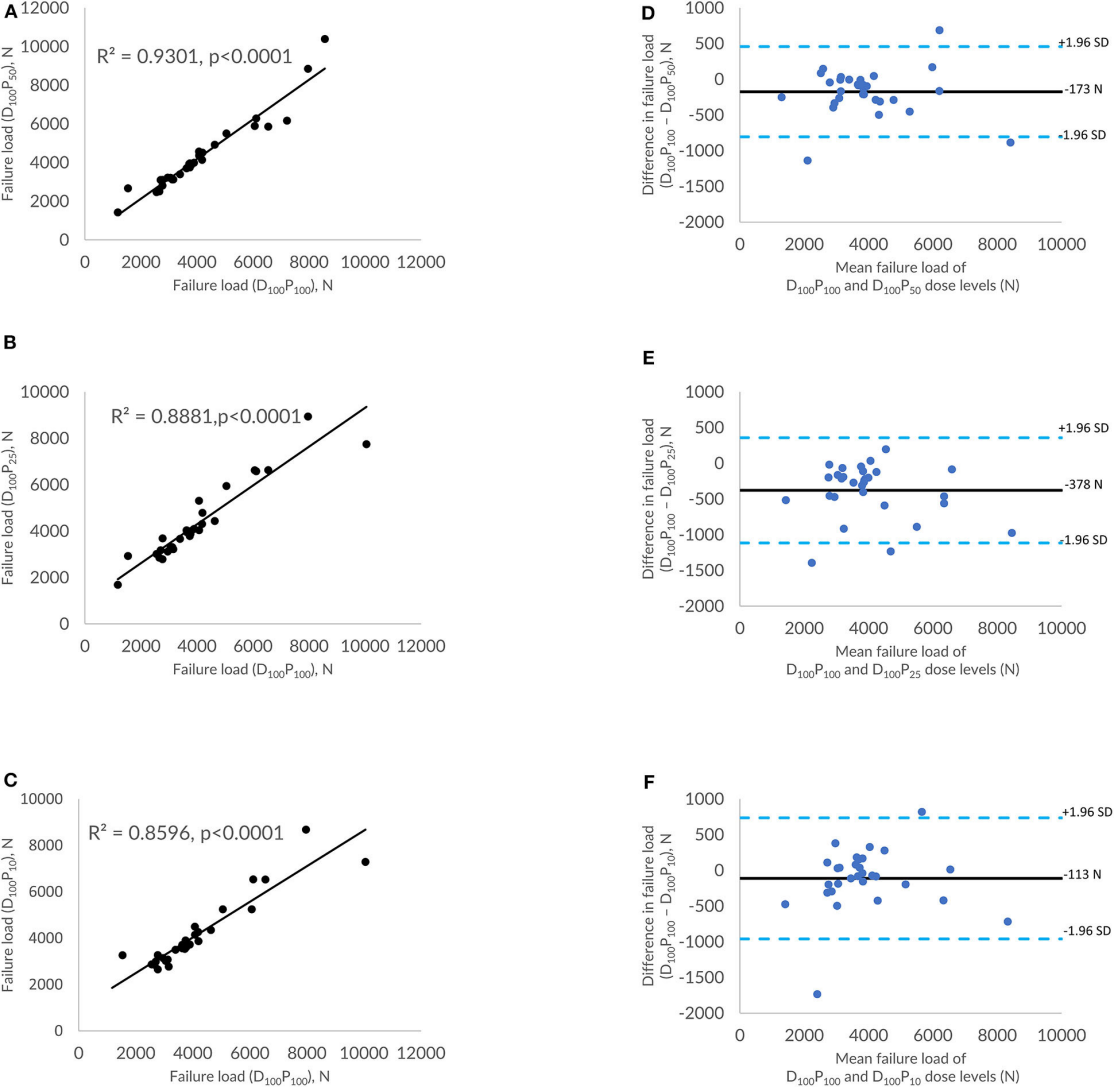
Effect of dose reduction through sparse sampling on FE-predicted failure load values. **(A)** Correlation plot between FE-predicted failure load values of original dose (100%) and 50% reduced dose, **(B)** correlation plot between FE-predicted failure load values of original dose (100%) and 75% reduced dose, **(C)** correlation plot between FE-predicted failure load values of original dose (100%) and 90% reduced dose, and **(D–F)** Bland-Altman plots representing the mean of FE-predicted failure load values vs. the difference between them. Horizontal lines represent mean and dashed line ±1.96 standard deviation. FL represents FE-predicted vertebral failure load (N).

Figures 3A–C shows the correlations between FE-predicted failure load values for the vertebral bone modeled from the low-dose images generated through the sparse sampling–based dose reduction. For the FE-predicted failure load, the *R*^2^ values amounted to 0.93, 0.89, and 0.86 for D_100_P_50_, D_100_P_25_, and D_100_P_10_, respectively. The effect of the sparse sampling–based dose-reduction method on the predicted failure loads was not significant (*p* > 0.05). To assess the relationship between FE-predicted failure loads obtained from the models of data acquired at different dose levels, we plotted the difference between failure loads of these three instances against the mean of them. Figures 3D–F shows the Bland-Altman plots of FE-predicted failure loads obtained from images at reduced dose through sparse sampling. A negative bias (173 N for D_100_P_50_, 378 N for D_100_P_25_, and 113 N for D_100_P_10_) toward the data obtained at full dose (D_100_P_100_) was evident in all the plots. Corresponding RMSCV (Table 2) values for D_100_P_50_, D_100_P_25_, and D_100_P_10_ were 8.6, 11.9, and 11.3%, respectively. The BMD values for D_100_P_50_, D_100_P_25_, and D_100_P_10_ dose levels is given in Table 3.

**Table 2:** Mean FE-predicted failure load, standard deviation, coefficient of correlation and RMSCV values for all the radiation dose levels.

		Failure load (N) ^*^	***R*^**2**^**	**RMSCV (%)**
Reference (Standard Dose)	D_100_P_100_	3,823 ± 1,423		
50% reduction	D_100_P_50_ [SS]	3,997 ± 1,426	0.93	8.6
	D_50_P_100_ [TC]	4,123 ± 1,335	0.96	12.06
75% reduction	D_100_P_25_ [SS]	4,201 ± 1,482	0.89	11.91
	D_25_P_100_ [TC]	4,735 ± 1,481	0.88	22.04
90% reduction	D_100_P_10_ [SS]	3,936 ± 1,382	0.86	11.38
	D_10_P_100_ [TC]	8,718 ± 5,201	0.43	54.19

*RMSCV, Root Mean Square Standard Deviation Coefficient Variation*.

*D, Dose level through tube current reduction, P= Dose level through sparse sampling*.

SS, Sparse sampling-based dose reduction method, TC= Tube current based dose reduction.

* *Represents mean ± SD*.

**Table 3:** Bone mineral density (BMD) measurements for vertebrae at different dose levels.

		**BMD - QCT (gm/cc)**		**Patient status**		
		**mean**	**Std. dev**	**Healthy (BMD>120)**	**Osteopenia (80 < BMD <120)**	**Osteoporotic (BMD <80)**
Full dose level	D_100_P_100_	89.30	14.55	1	9	2
Tube current reduction	D_50_P_100_	88.53	12.93	1	9	2
	D_25_P_100_	94.36	12.13	2	9	1
	D_10_P_100_	128.21	26.61	8	4	0
Sparse sampling	D_100_P_50_	94.82	17.30	1	9	2
	D_100_P_25_	88.34	14.28	1	9	2
	D_100_P_10_	90.08	12.42	1	8	3

*D, Dose level through tube current reduction; P, Dose level through sparse sampling*.

### Effect of Tube Current–Based Dose Reduction on FE-Predicted Vertebral Failure Load

**Figure 4:**
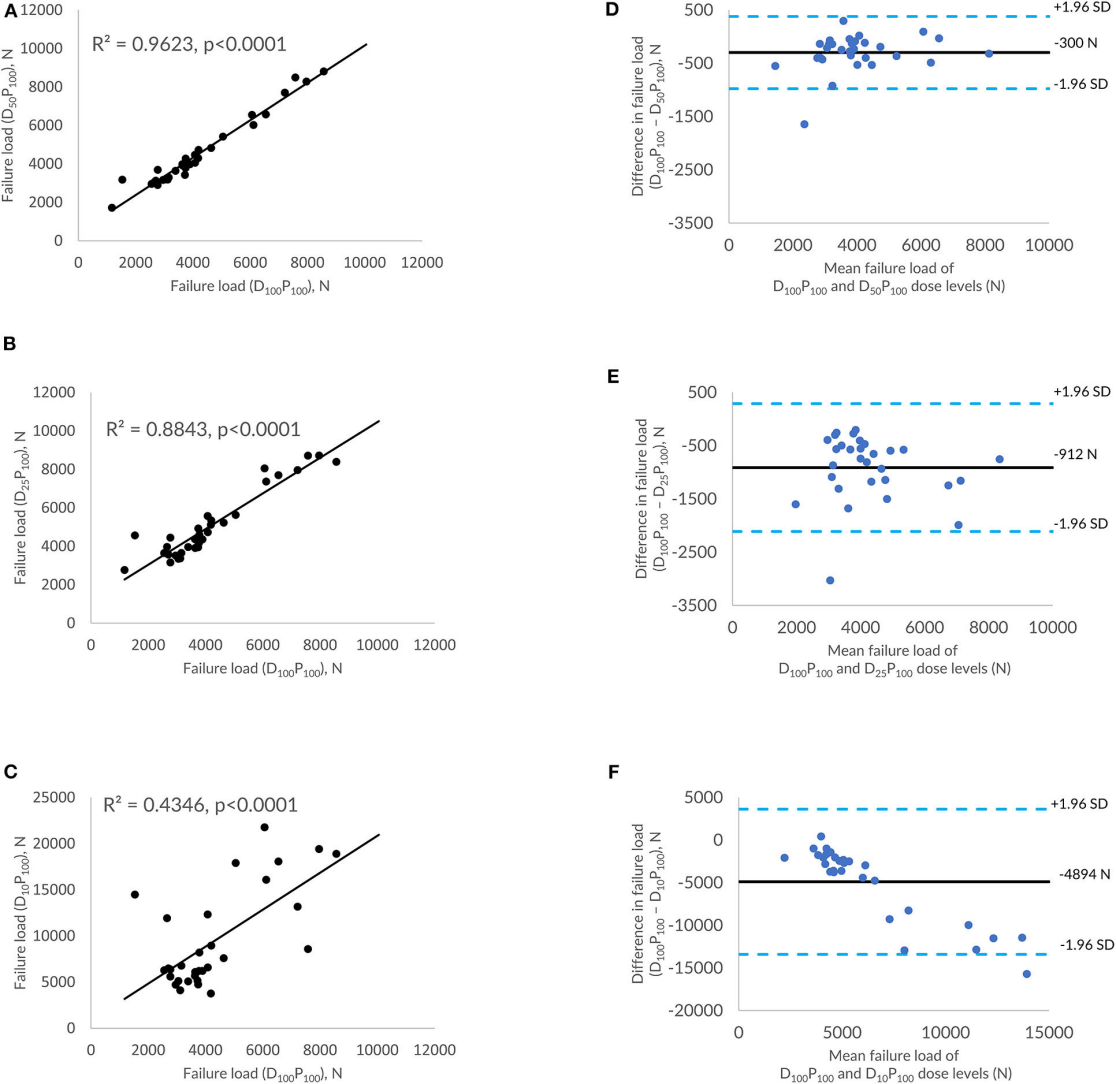
Effect of dose reduction through reduced tube current on FE-predicted failure load values. **(A)** correlation plot between FE-predicted failure load values of original dose (100%) and 50% reduced dose, **(B)** correlation plot between FE-predicted failure load values of original dose (100%) and 75% reduced dose, **(C)** correlation plot between FE-predicted failure load values of original dose (100%) and 90% reduced dose, and **(D–F)** Bland-Altman plots representing the mean of FE-predicted failure load values vs. difference between them. Horizontal lines represent mean and dashed line ±1.96 standard deviation. FL represents FE-predicted vertebral failure load (N).

Figures 4A–C shows the correlations between FE-predicted failure load values for the vertebral bone modeled from the low-dose images generated through tube current reduction–based dose reduction. *R*^2^ values amounted to 0.96, 0.88, and 0.43 for D_50_P_100_, D_25_P_100_, and D_10_P_100_, respectively. To assess the relationship between FE-predicted failure loads obtained from the models of data acquired at different dose levels, we plotted the difference between failure loads of these three instances against the mean of them. Figures 4D–F shows the Bland-Altman plots of FE-predicted failure loads obtained from images at reduced dose through tube current–based dose reduction. A negative bias (300 N for D_50_P_100_, 912 N for D_25_P_100_, and 4894 N for D_10_P_100_) toward the data obtained at full dose (D_100_P_100_) was evident in all the plots. Corresponding RMSCV (Table 2) values for D_50_P_100_, D_25_P_100_, and D_10_P_100_ were 12.06, 22.04, and 54.18%, respectively. The BMD values for D_50_P_100_, D_25_P_100_, and D_10_P_100_ dose levels is given in Table 3.

## Discussion

In the current study, we have evaluated the effect of sparse sampling and tube current reduction–based dose-reduction techniques on predicting vertebral bone strength using finite element analysis. Our results suggest that FE-predicted failure loads were not significantly affected by dose reduction through the sparse sampling method up to 90% (*R*^2^ = 0.93, RMSCV = 8.60% for 50%; *R*^2^ = 0.89, RMSCV = 11.90% for 75%; *R*^2^ =0.86, RMSCV = 11.30% for 90%) and 50% dose reduction through the virtually reduced tube current method (*R*^2^ = 0.96, RMSCV = 12.06%). However, any further reduction in radiation dose through the tube current reduction method shows considerable differences in FE-predicted failure load values with RMSCVs varying from 22.04 to 54.18%. Thus, the MDCT images obtained with a lower effective dose achieved through a sparse sampling–based method could be used for calculating FE-predicted vertebral strength to assess osteoporotic fracture risk.

The FE-predicted failure loads obtained from the models developed from the low-dose MDCT images acquired through the sparse sampling method showed high correlations (0.86 to 0.93) corresponding with the values obtained with the standard dose. The observed differences were low (8.60 to 11.30%) and are in line with the values reported in the literature (2.31 to 14.5%) (34–37). A much narrower band of differences are observed in the Bland-Altman plot (113 N to 378 N). It also showed a negative bias in predicted values. Previous studies have shown that sparse sampling–based dose reduction artificially increases the bone mineral density (BMD) value (up to 0.233 mg/cm^3^) (23), femoral neck BMD value (up to 0.006 g/cm^3^) (30), and femur bone strength (up to 120 N) (34). These results suggest that the FE-predicted failure load value for the models generated from the image data at reduced dose levels through sparse sampling may be higher than that generated using the standard dose. Corrections could be employed to account for the variation when using the value for diagnostic purposes or to extrapolate it for other analyses.

For MDCT images acquired at 50% reduced dose through tube current reduction, a strong correlation was observed between FE-predicted failure load values (*R*^2^ = 0.96, RMSCV = 12.06%). Further reduction in the radiation dose showed considerable differences in the failure load values as demonstrated in the Bland-Altman plots and the RMSCV values. The lower correlations showed that reduction in the dose significantly affected the image quality, in turn, affecting the FE analysis. In the FE analysis, material mapping relations are derived from the image pixel intensity (HU units), which directly affects the downstream analysis and calculation of the failure load. We also observed a negative bias toward the failure load values generated at standard radiation dose. Previous studies have shown that tube current reduction–based dose reduction method artificially increases the bone mineral density (BMD) value (up to 41.32 mg/cm^3^) (23) and the femoral neck BMD value (up to 0.276 g/cm^3^) (30) and femur bone strength (up to 1045 N) (34). Thus, we can conclude from these results that there is a slight increase in the value predicted from the image data acquired at a 50% reduction in dose level through tube current reduction than the images taken at the standard dose.

A sparse sampling–based dose-reduction strategy effectively produced high-quality image data, which resulted in stronger correlations between the failure loaded values derived from those image data acquired at a lower dose level (up to 90%) with those values obtained at a standard dose level. However, the same cannot be said to be true for the tube current–based reduction method, which shows only a 50% reduction is possible without compromising the accuracy of the predicted failure load value. In the literature, studies have shown that, compared to the MDCT images generated by tube current reduction–based dose reduction, MDCT images generated though the sparse sampling–based dose reduction method were superior in terms of image artifacts, contrast, and overall image quality (38). Furthermore, spine MDCT images generated through sparse sampling showed robust quantitative assessments of bone mineral density and microstructure compared to lower tube currents (23, 39). These results are consistent with the results observed in this study, comparing these two prominent dose-reduction strategies for predicting bone strength using finite element analysis.

Some limitations of the study must be taken into account when analyzing the results obtained. First, in the current study, we have analyzed the vertebrae only in the compression loading configuration for comparison purposes; however, when the model is simulated at different loading conditions such as flexion and rotation, the predicted failure loads may vary. Second, the number of subjects considered in the study is small (*n* = 12). Third, for a few subjects, large differences are observed in the predicted failure load values. These differences may be attributed to the observed higher material stiffness under all the dose configurations in those subjects. Fourth, the virtual low-dose simulations require knowledge about the CT system parameters to take into account the electronic readout noise. As this is scanner specific, different CT scanners may influence the low-dose simulations and consequently the impact on the FE analysis. Fifth, image reconstruction techniques can affect image quality and, in turn, affect the FE analysis. In the current study, the SIR method is used for reconstruction. If other reconstruction algorithms are used, the predicted failure loads may vary.

In conclusion, we have demonstrated the feasibility of using MDCT data generated at lower radiation dose, up to 90% through sparse sampling and 50% through reduced tube current for prediction of vertebral failure load using FE analysis with a minor correction to eliminate the bias. We have also shown that the sparse sampling–based dose-reduction method performs better than the tube current reduction method under low-dose conditions. Thus, we can conclude that there is a potential for using low-dose MDCT scans up to 50% dose level for predicting bone fracture risk and continuous osteoporosis monitoring with less radiation exposure.

## Data Availability Statement

The raw data supporting the conclusions of this article will be made available by the authors, without undue reservation.

## Ethics Statement

The studies involving human participants were reviewed and approved by Technische Universität München institute review board. Written informed consent for participation was not required for this study in accordance with the national legislation and the institutional requirements.

## Author Contributions

NR, MD, PN, KM, JK, TB, and KS contributed conception and design of the study. KS and TB supervised the work. NR performed the finite element analysis. KS and NR performed statistical analysis and wrote the first draft of the manuscript. All authors contributed to manuscript revision, read and approved the submitted version.

## Conflict of Interest

The authors declare that the research was conducted in the absence of any commercial or financial relationships that could be construed as a potential conflict of interest.
